# A Long-Awaited Structure Is Rev-ealed

**DOI:** 10.3390/v3050484

**Published:** 2011-05-05

**Authors:** Marie-Louise Hammarskjold, David Rekosh

**Affiliations:** Myles H. Thaler Center for AIDS and Human Retrovirus Research and The Department of Microbiology, University of Virginia, Charlottesville, VA 22908, USA

**Keywords:** HIV-1, Rev, RRE, RNA export, nuclear-cytoplasmic export, protein structure, RNA-protein binding, X-ray crystallography

## Abstract

It has been known for some time that the HIV Rev protein binds and oligomerizes on a well-defined multiple stem-loop RNA structure, named the Rev Response Element (RRE), which is present in a subset of HIV mRNAs. This binding is the first step in a pathway that overcomes a host restriction, which would otherwise prevent the export of these RNAs to the cytoplasm. Four recent publications now provide new insight into the structure of Rev and the multimeric RNA-protein complex that forms on the RRE [1–4]. Two unexpected and remarkable findings revealed in these studies are the flexibility of RNA binding that is demonstrated by the Rev arginine-rich RNA binding motif, and the way that both Rev protein and RRE contribute to the formation of the complex in a highly cooperative fashion. These studies also define the Rev dimerization and oligomerization interfaces to a resolution of 2.5Å, providing a framework necessary for further structural and functional studies. Additionally, and perhaps most importantly, they also pave the way for rational drug design, which may ultimately lead to new therapies to inhibit this essential HIV function.

It has been 25 years since the first publications established that the HIV *rev* gene is essential for HIV replication ([5,6]; for a review see [7]). The product of this gene, the Rev protein, binds to the Rev Response Element (RRE) [8–16], a multiple stem-loop RNA element, present in unspliced and incompletely spliced HIV mRNAs [17–19]. This binding is an essential step in a series of events that leads to the nucleo-cytoplasmic export of these mRNAs. Without Rev, they would be restricted by the cell from exiting the nucleus, because they retain introns [20,21]. Over the years, the study of Rev has led to important discoveries in the nuclear export field, including the discovery of the “leucine rich” nuclear export signal (NES) [22–25] and its binding partner, the export receptor Crm-1, also called Exportin-1 [26–29]. However, many of the structural details about Rev-RRE interactions have remained obscure, due primarily to a lack of structural information about Rev itself.

This situation has now changed with the appearance of a trio of papers from Alan Frankel’s lab [1–3], and one from a collaborative group of investigators at NIH and the Welcome Trust [4]. As a result of these publications, we now have a partial crystal structure for Rev, and an elegant structural model to describe how Rev binds to the RRE, oligomerizes, and forms the RNA-protein complex which serves as the export substrate for Crm-1.

Rev and RRE are HIV’s way of solving a host restriction that every retrovirus must overcome. This restriction arises because retroviral genetic organization makes the coding region for Gag and GagPol an intron that has to be removed to generate the spliced mRNA that expresses the envelope protein [30]. Thus, all retroviruses must export at least one mRNA from the nucleus that retains at least one intron. This RNA also serves as the genome to be packaged into new virus particles. In HIV, there is an additional second intron that roughly spans the envelope protein coding region, and alternative splicing generates multiple mRNAs for the accessory and regulatory proteins (including Rev) [31]. This complex RNA processing pattern brings about the necessity for HIV to export multiple mRNAs with retained introns.

It has been well established that mammalian cells do not normally allow mRNA with retained introns to reach the cytoplasm [20,21]. In the instances when such export does occur, special processes appear to be used. In some cases, this involves *cis*-acting RNA sequence elements (called CTEs) which link the intron-containing RNA directly to the Nxf1 mRNA export pathway [32,33]. This pathway is believed to be the major RNA export pathway used in mammalian cells and at least one retrovirus, Mason-Pfizer Money Virus, seems to have directly “stolen” this process from the cellular gene [34,35]. Other retroviruses, such as HTLV-I [36,37], MMTV [38], EIAV [39,40], and Jaagsiekte Sheep virus [41–43] employ viral specific components that work like Rev and RRE, but which show very little sequence similarity. These viruses, like HIV, thus use a virally encoded protein and a viral RNA adapter sequence to link mRNA export to the Crm-1 pathway. Recently the human foamy virus group of retroviruses has been shown to use a different mechanism to link export to Crm-1, relying on the cellular protein HuR protein as an RNA-binding adapter [44]. Why some viruses like HIV and HTLV evolved to use their own virally encoded proteins to link RNA export to the cellular Crm-1 export pathway remains a mystery.

For many years, a known feature of RNA binding by Rev has been the ability of the protein to form oligomers on a single RNA molecule [8,12,14,45–47] and several earlier studies have shown that Rev oligomerization is a necessary event for biological function. Some early reports suggested that Rev dimerizes first and then binds to the RRE [48,49], but there is now strong evidence that Rev recognizes its primary binding site as a monomer [50]. The RRE displays a complicated secondary structure within the HIV RNA that contains several “stems” and “loops [46,51]. The Rev primary binding site is formed by unusual features in the stem loop IIB region, including an A:G base pair and a distortion of the RNA helix [52,53]. The structure of this region of the RRE in complex with a peptide that mimics the RNA binding domain of Rev was determined some time ago. In this structure, the basic arginine-rich peptide inserts deeply into a wide RNA major groove, making specific contacts with the A:G base pair [54,55].

Two of the three papers by Daugherty *et al.* have now re-examined the nature and kinetics of Rev-RRE binding [1,2]. These studies show very clearly that assembly of the export complex is a well-orchestrated duet between RRE and Rev, involving the assembly of most likely six monomers of Rev on one RRE. This is a highly cooperative event, which relies upon the Rev protein’s RNA binding and oligomerization domains, as well as the RRE itself. The complex formed by this cooperative binding has an affinity that is 500-times greater than that of the tightest single interaction. What is surprising and marvelous about these events is that the Rev RNA binding domain shows amazing adaptability. It thus uses different surfaces of its arginine rich helical motif (ARM) to interact with the RNA, as the higher order complex gets assembled. The RRE also appears to be an active player in this assembly, as a single point mutation in stem loop IA, a site quite distant in the linear RRE sequence from the primary stem loop II B, was demonstrated to reduce the affinity of the complex five-fold.

The highly basic domain of Rev, and Rev’s tendency to oligomerize even in the absence of RNA, are at least partially to blame for the fact that it has taken so long to get a crystal structure. Preparations of Rev protein have usually demonstrated low solubility, with the uncontrolled formation of oligomers as well as insoluble aggregates whose formation are probably driven by its highly basic domain [56–58]. It is well known that previous Rev crystallization attempts have been the “graveyard” projects in many laboratories for many years. The keys to the recent structural advances are the clever ways in which the researchers involved have overcome these issues.

Previous genetic and biochemical studies had indicated that Rev oligomerization occurred through two different sets of hydrophobic sequences on different surfaces of the protein [59,60]. One set included Leu18 and Ile55, which were needed to form a cooperative RNA-Rev dimer, and the other involved Leu12 and Leu60, which were needed for higher order oligomerization. To weaken the higher order interaction, and prevent the problem of uncontrolled oligomerization *in vitro*, the Frankel group mutated Leu12 and Leu60 to Ser and Lys and removed 46 residues from the carboxyterminus of Rev [3]. Another trick to increase Rev solubility at high concentrations was to initially purify Rev as a fusion protein with the well-folded negatively charged B1 domain of streptococcal protein G (GB1) [2,61]. The GB1 domain was later cleaved off with a specific protease. The authors found that the GB1 fusion increased Rev solubility 100–1000 fold and they furthermore showed that potassium phosphate or sodium sulfate at 100 mM could maintain Rev in a soluble state upon removal of the fused GB1 domain. They argue that the acidic GB1 fusion or the oxyanionic counterions helped to solubilize Rev by charge neutralization, acting as RNA surrogates.

By following this protocol, Daugherty *et al.* obtained crystals that could be analyzed to a final resolution of 2.5Å [3]. Visible within the crystal lattice were the dimer interface of each asymmetric dimer unit, as well as the higher order oligomerization interface. Even though the oligomerization interface was mutated to allow crystallization, the Rev dimers in the crystal still packed against each other using the mutated interface. Just before this study appeared, another study by DiMattia *et al.* used Rev that was prepared in a complex with a specially engineered monoclonal Fab fragment [4]. This also overcame solubility issues and allowed crystallization, but the Fab fragment obscured the dimer interface and only the higher order oligomerization surface of Rev could be determined. The two higher order interface structures from both the Daugherty and DiMatta studies are nearly identical. As predicted, hydrophobic residues at position 12, 16 and 60 mediate the interaction.

So what does the structure of the first 70 amino acids of Rev look like, how does the dimer form and how does the higher order complex assemble? The Rev monomer folds as an antiparallel helix-loop-helix structure with the two helices in a nearly parallel orientation. It is stabilized by a series of hydrophobic interactions and electrostatic interactions between the two helices and adjacent residues. These interactions create a hydrophobic core that allows the monomer to dimerize by binding to the analogous core of a second monomer. Some of the residues involved in stabilizing the monomer, particularly Leu22 and Ile59 also stabilize the dimer. A diagram of this structure is shown in Figure 1, taken directly from Daugherty *et al.* [3].

The interaction between the hydrophobic core of each monomer positions them in a ‘V’ shape arrangement with a crossing angle of 120° (see Figure 1a). This arrangement predicts how the second monomer would contact the RNA outside of the primary binding site (see Figure 2). Interestingly, contact of the second monomer with RNA would use a different face of the helical ARM, rather than the one used to interact with the primary binding site. This model is consistent with the binding data already discussed above. Binding is cooperative and involves a protein-protein interaction as well as a protein-RNA interaction. At least two additional “V” shaped dimers of Rev also get recruited to the intact RRE in a cooperative fashion using the higher order interface interactions and further RNA binding.

Daugherty *et al*. point out that the inherent flexibility of this mode of RRE-Rev assembly, both in the way that the dimer forms and the way that the RNA is recognized, may be necessary to allow the Rev-RRE interaction to evolve and remain efficient as HIV sequence changes during the course of a natural infection [1].

This work is a long-awaited landmark achievement, as it clearly defines important structural features of the Rev-RRE complex. With the atomic structure of the Rev dimer and oligomer interfaces solved, and an increased understanding about how the RNA is recognized, the way is now open for rational drug design that could lead to compounds that would interfere with these interactions. However, many questions still remain about the recognition of the NES in the resulting complex. As Daugherty *et al.* themselves point out, steric hindrance would appear to not allow more than one or two molecules of Crm-1 to bind the complex. They suggest that the function of oligomerization may simply be to facilitate tight binding through cooperativity and that multimerization itself may not be used to recruit multiple Crm-1 molecules [3]. However, since the total number of Rev proteins that actually get recruited to an HIV mRNA remains an unanswered question, at this point it is difficult to say anything about the total number of Crm-1 molecules needed for export. In the end, the structural model thus sheds little light on how Rev-RRE binding eventually overcomes nuclear retention. The matter is further complicated by the fact that in the cell many other RNA binding proteins would also be expected to be bound to the RNA. Some of these are likely to be shuttle proteins, with their own NES sequences. Thus, the details of how Rev/RRE binding actually promotes export, is a still an unsolved mystery.

## Figures and Tables

**Figure 1:**
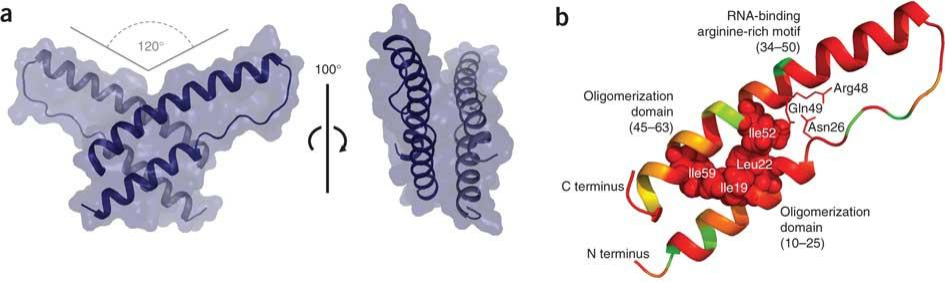
Structure of the Rev Dimer. (**a**) Two views of a surface representation of dimer are shown. (**b**) The folded core of a Rev monomer with its functional domains highlighted is shown. The amino acids prominent in mediating the core structure are specified. The different colors of the ribbon indicate amino acid conservation among 1201 HIV-1 isolates in the Los Alamos Sequence Database, with green representing the least conservation (26%) and red the highest (100%). Reprinted with permission from Macmillan Publishers Ltd: *Nature Structural & Molecular Biology* 17, 1337–1342 (2010) [3].

**Figure 2:**
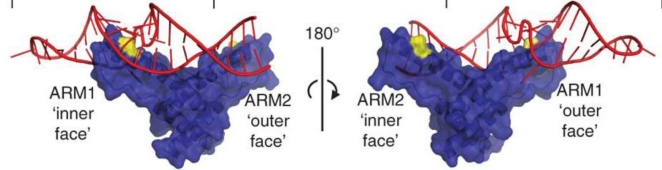
Model of the Rev dimer interacting with a model Rev Response Element (RRE) stem IIB binding site. In this model, the second monomer binds cooperatively using a different surface of the arginine rich helical motif (ARM) to contact the RNA (red). The yellow residue in the diagram represents Asn40 which contacts the RNA on the “inner face” of ARM1 but not ARM2 which uses the “outer face” of the ARM to bind. Reprinted with permission from Macmillan Publishers Ltd: *Nature Structural & Molecular Biology* 17, 1337–1342 (2010) [3].
